# The Association between Medicare Advantage Market Penetration and Diabetes in the United States

**DOI:** 10.3389/fpubh.2015.00229

**Published:** 2015-10-08

**Authors:** Steven W. Howard, Stephanie Lazarus Bernell, Jennifer Wilmott, M. Faizan Casim, Jing Wang, Lindsey Pearson, Caitlin M. Byler, Zidong Zhang

**Affiliations:** ^1^Health Management and Policy, Center for Outcomes Research, Saint Louis University, St. Louis, MO, USA; ^2^Health Policy and Management, School of Social and Behavioral Health Services, Oregon State University, Corvallis, OR, USA; ^3^Biostatistics, Saint Louis University, St. Louis, MO, USA; ^4^HealthCom Research and Solutions, Inc., Fredericksburg, VA, USA; ^5^Saint Luke’s Health System, Kansas City, MO, USA; ^6^The University of Texas MD Anderson Cancer Center, Houston, TX, USA; ^7^Jefferson County Health Department, Hillsboro, MO, USA

**Keywords:** cardiometabolic conditions, diabetes, cardiovascular disease, MEPS, Medicare Advantage, market penetration, spillover effects

## Abstract

The objective of this study is to explore the extent to which managed care market penetration in the United States is associated with the presence of chronic disease. Diabetes was selected as the chronic disease of interest due to its increasing prevalence as well as the disease management protocols that can lessen disease complications. We hypothesized that greater managed care market penetration would be associated with (1) lower prevalence of diabetes and (2) lower prevalence of diabetes-related comorbidities (DRCs) among diabetics. Data for this analysis came from two sources. We merged Medicare Advantage (MA) market penetration data from the Centers for Medicare and Medicaid Services (CMS) with data from the Medical Expenditure Panel Survey (MEPS) (2004–2008). Results suggest that county-level MA market penetration is not significantly associated with prevalence of diabetes or DRCs. That finding is quite interesting in that managed care market penetration has been shown to have an effect on utilization of inpatient services. It may be that managed care protocols do not offer the same benefits beyond the inpatient setting.

## Introduction

Over the past 15 years, there has been a marked increase in the number of Medicare beneficiaries enrolled in managed care plans. Medicare Advantage (MA) enrollment among Medicare beneficiaries has rapidly increased from 9% in 1995 to 30% in 2014 (1, 2). The market penetration rate of MA varies from state to state and county to county, with urban areas having higher enrollment rates (26%) compared to rural areas (15%) (3, 4).

Research on the effects of managed care penetration has primarily focused on inpatient care and prevention services. Results indicate that increased managed care market penetration is linked to higher rates of vaccinations and disease screenings among the general population (5–7). Greater managed care market penetration is also associated with reduced utilization of unnecessary inpatient procedures among Medicare beneficiaries (8, 9) reductions in inpatient complications (10) and reduced mortality rates after hospital discharge (11, 12).

The spillover effect of managed care processes is also of great interest. Work in this area suggests that strategies implemented by health care providers contracted with managed care organizations (MCOs) spillover to those providers’ patients who are not members of MCOs, particularly in areas of high MCO market penetration (5, 6, 9, 10). If spillover effects are truly impactful, it is reasonable to posit that in areas with high MCO market penetration, physicians will identify chronic conditions more quickly and prescribe treatment (and disease management), which may lead to slower progression of disease (5, 9, 13–17).

The objective of this study is to explore the extent to which managed care market penetration in the United States is associated with the presence of chronic disease. Diabetes was selected as the chronic disease of interest due to its increasing prevalence as well as the disease management protocols that can lessen disease complications. We hypothesized that greater managed care market penetration would be associated with (1) lower prevalence of diabetes and (2) lower prevalence of diabetes-related comorbidities (DRCs) among diabetics.

Diabetes mellitus (DM) affects nearly 29.1 million (9.3%) people in the United States, and costs the nation an estimated $245 billion annually (18). More than one in four Americans over the age of 65 have DM, and their morbidity contributes to the financial strain on the Medicare and Medicaid programs (18, 19).

Improperly treated DM can result in elevated risk for other DRCs, including vascular disease, neuropathy, renal disease, retinopathy, and cardiovascular complications, such as stroke, heart disease, coronary artery disease, and myocardial infarction. Compared to a non-diabetic, an individual with DM has a two to fourfold greater risk of dying from heart disease or having a stroke (18).

Type 2 diabetes is often used as an example of a chronic condition that can benefit from disease management tools often used by managed care (20–22). When effectively implemented, disease management protocols have reduced the rate of diabetes and associated DRCs (23–30).

## Materials and Methods

The data for this study were drawn from the Agency for Healthcare Research and Quality’s (AHRQ) Medical Expenditure Panel Survey (MEPS) (31) and the Centers for Medicare and Medicaid Services (CMS). CMS provided the MA market penetration data (32, 33). MA market penetration is calculated by taking the number of MA enrollees in a county or state divided by the number of Medicare beneficiaries in the same area. We merged MA market penetration data with data for all Medicare beneficiaries in MEPS from 2004 to 2008 (panels 9–12), based on each beneficiary’s county of residence.

The MEPS household component (MEPS-HC) is a nationally representative survey of non-institutionalized individuals residing in households sampled from the previous year’s National Health Interview Survey. The individuals followed in MEPS are grouped into panels and surveyed five times (rounds) over 2 years. A new panel begins each year, resulting in the overlapping panel design (one panel’s first year is concurrent with the previous panel’s second year) (31). After university institutional review board approval, we used data from the non-public MEPS-HC (via AHRQ’s secure data center), including information on individuals’ and households’ demographics (including county and state of residence), diagnoses, and coverage by Medicare and other insurance.

In addition to standard exploratory data analysis, two multivariate logistic regression models were used to explore the association between MA market penetration, diabetes, and DRCs at the individual level. The key independent variable of interest, county-level MA market penetration, was categorized into three groups: <12.5, 12.5–24.9%, and ≥25%. In establishing these groupings, we considered previous research by Baker et al. (5) and Bundorf et al. (8) with upward adjustment of the lower and middle categories to account for the growth in MA market penetration nationwide since the date of these publications.

Individual-level characteristics include race, gender, age, level of education, income as a percent of federal poverty level (FPL), urban residence (in a metropolitan statistical area), having other insurance in addition to Medicare, and having a managed care plan (34, 35).

In the first model, exploring the relationship between diabetes, DRCs and MA market penetration, we retained the full sample of 8089 Medicare beneficiaries in the 2004–2008 MEPS (panels 9–12). The second model evaluates the association between MA market penetration and the prevalence of DRCs *among* diabetic beneficiaries. In this model, individuals were selected who reported diabetes at the end of the first (*n* = 1761) or second (*n* = 1926) years of their panel. DRCs were defined using the priority conditions in MEPS and included hypertension, coronary heart disease (CHD), stroke or transient ischemic attack (TIA), high cholesterol, diabetes-related eye or kidney disease, angina, or heart attack (36, 37).

Survey procedures were used to allow for the complex sampling design of MEPS. We used weights provided by MEPS to ensure that the data were representative of the US civilian, non-institutionalized Medicare population at the time the data were collected. The weights were applied in both the descriptive analysis and the logistic regression model, to adjust for non-response and attrition, and in order to make estimates of person-level changes in selected variables.

## Results

### Descriptive Analysis

The population for this study included 8089 Medicare beneficiaries who participated in MEPS between 2004 and 2008 (panels 9–12). The data had roughly equal representation from each of the four panels. Women made up 56.6% of the sample, and 53% were married (Table 1). Nearly 85% were identified as White, and only 7% were Hispanic. Nearly one in five were under the age of 65 (18.8%), which is slightly more than the national average (17%), and one in five were at least 80 years of age. One-third resided in a household with income higher than 400% of FPL. While more than 25% did not receive a high-school education, 20% of the sample had a college education or greater. Nearly 80% of respondents lived in urban areas (as defined by the US Census Bureau). Slightly more than half had some insurance coverage besides Medicare. Fourteen percent had coverage through MCOs. Among Medicare beneficiaries, one in four resided in counties with high MA market penetration (≥25%), and one in six resided in areas of moderate MA market penetration (12.5–25% county penetration).

**Table 1:** **Descriptive statistics**.^b^

Variable	*N*	Percent	Variable	*N*	Percent
**Age groups**			**Education**		
<50	612	6.48%	Less than high school	2715	25.86%
50–64	1123	12.40%	High school	2613	35.48%
65–69^a^	1922	24.14%	Some college	1282	18.42%
70–74	1566	19.27%	4 years college	744	11.40%
75–79	1302	17.07%	>4 years college^a^	579	8.84%
80–84	936	12.40%	**Marital status**		
>85	628	8.24%	Married^a^	4068	53.00%
**Gender**			**Managed care organization**		
Female^a^	4616	56.62%	Had MCO at the end of first year^a^	1268	14.14%
**Race**			**Other insurance**		
White^a^	6287	84.77%	Had other insurance^a^	3574	51.27%
**Hispanic**			**MA market penetration**		
Not Hispanic^a^	7032	92.80%	<12.5%	4741	57.70%
**Income (% FPL)**			12.5 to <25%	1218	16.47%
<100% FPL	1535	13.54%	25% or more^a^	2130	25.83%
100–125%	684	6.84%			
126–200%	1458	17.27%	**Panel**		
201–399%	2186	29.02%	Panel 9 (2004–2005)^a^	2017	24.37%
>400% FPL^a^	2222	33.33%	Panel 10 (2005–2006)	2115	24.37%
**Urban/rural**			Panel 11 (2006–2007)	2283	25.30%
Urban^a^	6248	79.28%	Panel 12 (2007–2008)	1674	25.96%

*^a^Reference groups*.

*^b^All percent statistics are weighted. N is unweighted*.

### Multivariate Logistic Regression Results

The main independent variable of interest is MA market penetration. The results suggest that market penetration of managed care (in the respondent’s county of residence) is not significantly associated with individual-level diabetes or DRCs. Respondent’s insurance type (private insurance in addition to Medicare, and/or more specifically managed care membership) is also not significantly associated with diabetes or DRCs (Table 2).

**Table 2:** **Odds ratios**.^a^

Variables	Odds ratios	
	Prevalence of diabetes and diabetes-related comorbidities (DRCs) (*n* = 8089)	Prevalence of DRCs among diabetics (*n* = 1926)
Market penetration <12.5%	1.19	0.996
Market penetration 12.5 to <25%	9.23	1.662
Non-White	1.258**	1.878
Hispanic	1.181	1.26
Federal poverty level (FPL) <100%	1.312∗	0.964
FPL 100–125%	0.986	0.927
FPL 126–200%	1.263∗	2.267
FPL 201–399%	1.234∗	1.334
Not married	0.987	1.47
Male	0.94	0.737
Less than high-school degree	1.195	1.541
High-school degree	1.162	0.722
Some college	1.199	1.414
4 years of college	0.975	0.663
Age < 50	0.241***	0.478
Age 50–64	1.084	0.988
Age 70–74	1.539***	0.859
Age 75–79	1.732***	0.968
Age 80–84	1.625***	1.836
Age 85+	1.696***	1.167
No MCO	1.028	1.192
No other insurance	0.933	0.647
Not in MSA	0.787**	2.867**

*p < 0.10; **p < 0.05; ***p < 0.01.

*^a^All statistics are weighted*.

Factors that appear to be significantly associated with diabetes and DRC prevalence include race, reduced income, age, and place of residence. The only variable in the weighted, multivariate model that is significantly associated with DRCs among diabetics is place of residence. Living in a rural area is significantly associated with a reduced likelihood of diabetes and DRCs among the overall MEPS Medicare beneficiary group.

However, rurality is strongly associated with an increased likelihood of DRCs *among* diabetics. It may be that rural diabetics have reduced access to specialty care, resulting in a greater likelihood of DRCs.

The odds of having diabetes or a DRC increase with age and for non-White individuals. Compared to respondents with household incomes ≥400% of FPL, a lower level of income increases the odds of diagnosis. Although a significant relationship was observed between diagnosis and income, no statistically significant relationship was observed between diagnosis and education.

Our analysis relies on publically available data and uses fairly straightforward methods. It may be the case that in order to tease out the effect that MA market penetration has on diabetes a different type of empirical analysis is needed. To this end, we developed an alternative model that examined the association between MA market penetration and the occurrence of new cases of diabetes among Medicare beneficiaries between year 1 (*n* = 1761) and year 2 (*n* = 1926). We excluded 1761 respondents who reported having diabetes at the end of their first year in the MEPS panel. This resulted in retention of 6328 (79.2%) of our original observations. Like the other models in Table 2, the managed care market penetration variable was not a significant factor in the likelihood of being newly diagnosed with diabetes (results not shown in this paper). The one interesting finding from this additional model was that compared to beneficiaries between the ages of 65 and 70, respondents age 85 or older had less than half the odds of being diagnosed with diabetes. This lower incidence of diabetes in the oldest group of Medicare beneficiaries may be a survivor effect (38, 39).

## Discussion

In this study, we examined the associations between MA market penetration and Medicare beneficiaries’ (1) diabetes and DRC diagnoses overall and (2) DRCs among diabetics. This analysis is one of the first since the passage of the Medicare Modernization Act to address the role that market penetration of MA may have on chronic disease and disease progression.

Although the empirical results do not support our hypotheses, this study is important on two fundamental levels. The first relates to whether “non-significant results” can tell a story. While our results are not statistically significant, that finding in and of itself is *substantively* significant. It is possible that the lack of association between market penetration and diabetes is an indication that managed care is not working as well as previous research has indicated. It could also be the case that there are limited spillover effects of managed care. Lastly, it could be that the protocols are so varied in scope and implementation that no systematic impact can be detected (i.e., more direct measures of the type and longevity of disease management protocols within the individual plans). The second contribution is methodological. We suggest researchers use our study as a springboard for future work.

### Limitations

While this study had unique strengths, there were limitations. Screening rates influence the prevalence rates of diagnosed disease. Diabetes screening rates for this population were unavailable. Since screening rates are known to increase under managed care (5–7), we expect that there is higher prevalence of screening in areas of higher managed care market penetration, all other things being constant. In the absence of a system-wide model, the effect of changes in screening procedures and rates on diabetes incidence and complications cannot be fully ascertained.

We found significant results for rural Medicare beneficiaries. However, given that most rural areas in the United States are primary care-shortage areas, it is possible that the rate of *undiagnosed* diabetes and DRCs is higher in rural areas, and the prevalence of *diagnosed* disease lower. The modest number of rural Medicare beneficiaries in MEPS makes it difficult to understand the impact of managed care market penetration. Future work will include a larger population of rural individuals, and will focus on the relationships between rurality and chronic disease.

The MEPS survey data are self-reported. With self-report data, MEPS is subject to recall error and the honesty of participants’ responses to surveyors.

At a minimum, new work on the effects of managed care (or specifically MA) market penetration on chronic diseases, such as diabetes, should include the following:

(1) Representative data from all United States counties. In our study, only a sample of United States counties is represented in each MEPS panel. If these counties (or those not sampled) are outliers on the distribution of MA market penetration, this study’s findings may not be indicative of the true national trends.
(2) Clinical data. Due to data limitations, our study relied on the use of household-reported health information, not clinical data. Clinical or medical claims data may reduce any problems of recall bias and provide more precise diagnoses. In addition, clinical data may be able to capture differences in clinical severity of the underlying diagnoses and enable researchers to consider the individual’s overall “disease portfolio.”
(3) At least 4 years of longitudinal data. In our study, disease diagnosis variables were limited primarily to the priority conditions questions in MEPS, which are asked only at the end of each year (rounds 3 and 5), leaving only 1 year for new diagnoses to be reported.

On balance, we do not take our results as the definitive answer to the prevailing question: Does MA market penetration impact disease progression? Given that MA market penetration is at 30%, and growing each year, future work in this area is essential (40). The Affordable Care Act and various state reform efforts call for innovative healthcare delivery models that bear similarities to managed care. Understanding the role of MA market penetration (and managed care more broadly) is critical given that the new delivery models are intended to improve population health outcomes and reduce the incidence of chronic diseases.

## Conflict of Interest Statement

The authors declare that the research was conducted in the absence of any commercial or financial relationships that could be construed as a potential conflict of interest.
